# Chest Exercises: Movement and Loading of Shoulder, Elbow and Wrist Joints

**DOI:** 10.3390/sports10020019

**Published:** 2022-01-31

**Authors:** Pascal Schütz, Pia Zimmer, Fabian Zeidler, Michael Plüss, Katja Oberhofer, Renate List, Silvio Rene Lorenzetti

**Affiliations:** 1 Institute for Biomechanics, ETH Zurich, Leopold-Ruzicka-Weg 4, 8093 Zurich, Switzerland; ps@ethz.ch (P.S.); pia.zimmer@outlook.com (P.Z.); fabian.zeidler@chello.at (F.Z.); michael.pluess@hest.ethz.ch (M.P.); rlist@ethz.ch (R.L.); 2 Department of Medicine, Sports and Healthcare, University of Applied Sciences Technikum Wien, Höchstädtplatz 6, 1200 Vienna, Austria; 3 Swiss Federal Institute of Sport Magglingen (SFISM), Hauptstrasse 247, 2532 Magglingen, Switzerland; katja.oberhofer@baspo.admin.ch; 4 Human Performance Lab, Schulthess Clinic, Lengghalde 2, 8008 Zurich, Switzerland

**Keywords:** shoulder, strength exercise, pectoralis training, kinetics, kinematics

## Abstract

Injuries to the shoulder are very common in sports that involve overhead arm or throwing movements. Strength training of the chest muscles has the potential to protect the shoulder from injury. Kinematic and kinetic data were acquired in 20 healthy subjects (age: 24.9 ± 2.7 years) using motion capture, force plates for the bench press exercises and load cells in the cable for the cable pulley exercises with 15% and 30% of body weight (BW). Joint ranges of motion (RoM) and joint moments at the shoulder, elbow and wrist were derived using an inverse dynamics approach. The maximum absolute moments at the shoulder joint were significantly larger for the cable pulley exercises than for the bench press exercises. The cable cross-over exercise resulted in substantially different joint angles and loading patterns compared to most other exercises, with higher fluctuations during the exercise cycle. The present results indicate that a combination of bench press and cable pulley exercises are best to train the full RoM and, thus, intra-muscular coordination across the upper limbs. Care has to be taken when performing cable cross-over exercises to ensure proper stabilisation of the joints during exercise execution and avoid joint overloading.

## 1. Introduction

Strength training plays an integral part in the prevention of and rehabilitation from injury, as well as improvement of sports performance [1]. Thereby, the primary goal of strength training is to increase muscular strength for stabilising the joints while simultaneously improving inter- and intra-muscular coordination [2]. Focusing on the shoulder joint and upper limb, injuries are very common in sport disciplines that involve overhead arm or throwing movements, such as tennis, baseball or basketball [3]. Here, well-trained chest muscles may help to reduce injury risks by better stabilising the shoulder joint and the interconnected upper limbs [4].

Bench press exercises are the most popular strength exercises for developing upper body strength, especially of chest muscles [5]. The core muscle groups which are trained during bench press exercises are the pectoralis major, the triceps brachii, the anterior deltoid and the medial deltoid, serving as key stabilisers of the shoulder joint [1]. Another common method to improve chest muscle strength is to perform strength exercises on a cable pulley system [6]. The cable pulley system offers a wide range of exercise execution types that can be adapted to individual preferences and requirements. From a biomechanical point of view, the direction of resistance during bench press exercises is always vertical due to gravity, while the resistance during the cable pulley exercises is in the direction of the cable.

While a high number of studies have analysed muscle activity patterns during bench press exercises [7,8,9,10,11,12,13], relatively little research has been done on joint kinematics and kinetics of the upper limbs for both bench press and cable pulley exercises. The authors of [14] investigated the effects of exercise intensity on trunk muscle activity during pulley-based shoulder exercises on an unstable support surface. The results did not show any significant changes in muscle activation patterns for different exercise intensities. In a different study, it was found that the shear forces in the shoulder joint were more constant, and the joint ranges of motion (RoMs) were larger using a variable resistance machine compared to a cable pulley system [6]. More recently, ref. [15] compared the kinematics and kinetics of the upper limbs during external and internal rotation exercises of the shoulder with constant versus elastic resistance. While the joint RoM did not differ significantly between the two resistance types, shoulder joint loading was found to be significantly higher with constant resistance. No study was found comparing upper limb kinematics and kinetics for different strength exercises that specifically target the stabilisation of the shoulder joint.

It was previously found in professional baseball pitchers that present weakness of shoulder strength is associated with increased risk of throwing-related injuries, stressing the importance of targeted muscle strengthening plans for injury prevention [4]. While strength training can have a beneficial effect on joint function and sports performance, there is also a reported risk of injury due to overstressing or incorrect exercise execution [16]. The high injury risks of the shoulder, both during sports performance and strength training, demands an improved understanding of joint kinematics and kinetics during non-fatiguing exercise executions to develop safe and effective training guidelines. Therefore, the aim of the present study was to compare the kinematics and kinetics of the upper limbs during flat and inclined bench press exercises, as well as during two different cable pulley exercises, namely the cross-over exercise, also known as butterfly, and the pull-over exercise at moderate intensity. Specifically, the RoMs of the wrist, elbow and shoulder joint, as well as the maximum joint moments during each repetition cycle, were analysed and compared between the different types of exercises and two different load magnitudes.

## 2. Materials and Methods

### 2.1. Participants

Kinematic and kinetic data were acquired in 10 healthy male and 10 healthy female subjects (age: 24.9 ± 2.7 years, height: 175.2 ± 9.0 cm, weight: 68.6 ± 11.1 kg). The sample size of *n* = 20 was in line with similar studies that compared joint kinematics between different types of strength exercise (i.e., *n* = 15 in [17] or *n* = 12 in [15]). Inclusion criteria were an age between 18 and 45 years and experience with weight training, with at least two hours per week of training for a sufficient length of time to be familiar with the selected exercises. Exclusion criteria were current injury or illness, previous surgery to the shoulder or upper limbs, neurological disorders or current medical treatment. The study was approved by the ethics committee of the ETH Zurich, Switzerland (2017-N-46). All participants signed a declaration of consent to participate in the study.

### 2.2. Experimental Approach

Prior to data acquisition, each participant conducted a specific, five-minute warm-up session using sets of the exercises with minimal loading. Each subject received precise instructions on how to execute the exercises (i.e., bench press flat, bench press inclined, cable cross-over, cable pull-over, see Additional File 1). For the bench press exercises, a weight bench with an adjustable backrest, together with a barbell with a tare weight of 10 kg and variable weight plates, were used. Grip width for the bench press exercises was defined as the length of the upper arm times two, plus once the shoulder width. This is the standardised definition of grip width in weight lifting [18]. Cable pulley exercises were performed on a cable pulley system with two separate towers that were height-adjustable. For the cross-over exercise, both towers were used with a single-hand grip on either side, and the loading was evenly divided between both towers. The pull-over exercise was executed using just one tower with a nylon rope with a rubber-end stop and swivel to hold with both hands. Each exercise was performed with two different loading conditions that were 15% and 30% of the subject’s body weight (BW).

Following the warm-up session, each participant performed the flat and inclined bench press exercises, as well as the cross-over and pull-over exercises according to the given guidelines. The order of the exercises was chosen randomly for each subject, but each exercise was performed first with a loading of 15% BW, directly followed with a loading of 30% BW. Six consecutive repetitions were recorded for each exercise and both loading conditions. Between each set of six repetitions, the subjects took a break of at least 2 min to recover and avoid muscular fatigue. Focus was given to correct exercise execution (i.e., joint alignment) according to guidelines rather than speed of execution and/or the lifting of maximum weight.

### 2.3. Data Acquisition

To collect kinematic data of the upper extremities in all three planes of motion, a Vicon MX40 system (Vicon Motion Systems Ltd., Oxford, UK) with 22 cameras was used. The resolution of each camera was 2353 × 1728 pixels, capturing at a frequency of 100 Hz. A skin marker set with a total of 54 markers was used, which was specifically designed for evaluating the biomechanics of the upper limbs and previously applied to strength training research [15]. A cluster of at least four markers was used to define each body segment. The marker set by [15] was supplemented with four additional markers on the trunk, because the ones on the spine and scapula could not be used during the bench press trials. Markers were positioned on anatomical structures with little skin movement and high visibility throughout the whole exercise cycle. Markers had a diameter of 14 mm, except the markers on the hands, on the highest point of the sternum and on the sternoclavicular joints, which had a diameter of 9 mm. Two Kistler force plates (Kistler Group, Winterthur, Switzerland, Type 9281B, width and length of 400 × 600 mm), which were embedded in the floor of the movement analysis laboratory, were used to record the ground reaction forces during the bench press exercises. For the kinetic measurements of the cable pulley exercises, two 1 kN load cells operating at 2000 Hz (SM-1000N, Interface Inc., Atlanta, GA, USA) were placed in series between the handle and the cable. Additionally, optical markers were placed on the cable pulley handles and six additional markers on the cable in order to assess the direction of the external force due to resistance during the pull-over and cross-over exercises. The recorded movement trajectories of these markers were also used to separate the exercise cycles into individual repetitions. Optical markers were placed on each end of the barbell to separate the cycles for the bench press exercises.

### 2.4. Data Processing and Analysis

The marker trajectories from optical motion capture were tracked and labelled using Vicon Nexus 2.4 (Vicon Motion Systems Ltd., Oxford, UK) and, subsequently, exported for the analysis of joint kinematics using Matlab 2014 (Mathworks, Natick, MA, USA). Exercise cycles were separated into individual repetitions using the mean values of the z coordinates of the markers on each handle, with a minimum velocity of 30 mm/s to indicate movement for the bench press exercises and 40 mm/s for the cable pulley exercises, respectively. The positions of the glenohumeral joint centre (i.e., shoulder joint), elbow joint centre and wrist joint centre were functionally derived based on the kinematic data from nine basic motion tasks, previously introduced by [15] and described in Additional File 2. Orthogonal and right-handed segmental coordinate systems were defined based on the position of the joint centres and the segmental markers.

The kinematic and kinetic data from the first repetition of each exercise cycle were excluded from further analysis. Thus, five repetitions of each exercise for the right and left extremity of each subject were further processed, making a total of 200 evaluated cycles. The positions and orientations of the segments during each repetition were determined using a least-squares fit of the corresponding marker clusters [19]. Upper limb joint kinematics were derived from the segmental positions using the joint coordinate system convention created by [20] and recommended by the International Society of Biomechanics (ISB) [21]. The movement of the shoulder joint was simplified, described as the relative motion of the upper arm with respect to the torso, similar to [17], not considering the shoulder girdle as individual segments due to well-known skin movement artefacts across the clavicle and scapula [22]. Details on the definitions of segmental and joint coordinate systems are given in the Additional File 3. It is important to note that the joint coordinate systems based on the convention by [20] are non-orthogonal, depending on the position and orientation of the adjacent segments with respect to each other.

The joint moments (M_abs_) and their maximum (M_max_) at the shoulder, elbow and wrist during each repetition cycle were calculated using a quasi-static inverse dynamics approach based on the positions and orientation of the segments, the measured external forces and the gravitational force of the segments and handle [17,23]. The data from the force sensors were filtered using a third-order low pass Butterworth filter with a cut-off frequency of 70 Hz. Due to the different recording frequencies of the kinetic and kinematic data, the force sensor data were down-sampled to 100 Hz. The BW of each subject was subtracted from the total force vector during the bench press exercises to ensure that only the mass and accelerations of the moved segments and the barbell had an impact on the calculation of the joint moments. The centre of mass (CoM) and mass of the moved segments were calculated according to [24]. The mass of the handle was 0.07 kg for the pull-over exercise and 0.05 kg each for the cross-over exercise, and its centre of gravity was assumed to be equal to the CoM of the hand. The direction of the force vector during the cable pulley exercises was calculated using a least-squares fit of the line between the six markers attached to the cable.

Joint angles and joint moments were resampled over time, and joint moments were additionally normalised to BW. Mean maximum and mean minimum joint angles, as well as mean maximum joint moments (M_max_), were calculated across all repetition cycles of each exercise (i.e., bench press flat, bench press inclined, cable cross-over, cable pull-over). Joint RoMs were calculated as the difference between the maximum and minimum joint angles.

### 2.5. Statistical Analysis

Statistical analysis was carried out using IBM SPSS Statistics 24 (SPSS AG, Zurich, Switzerland) software. The independent variables were the four different types of strength exercises (i.e., bench press flat, bench press inclined, cable cross-over, cable pull-over) and the two magnitudes of externally applied loads (i.e., 15% and 30% BW). At the wrist and the elbow joint, mean RoMs and mean M_max_ in the sagittal and frontal plane and transversal plane, respectively, were statistically compared between exercises and applied loads. At the shoulder joint, only the absolute M_max_ values were statistically compared due to the complexity of the shoulder joint in 3D [17].

Prior to statistical analysis, outcome variables were checked for normal distribution using Q–Q plots. Given normal distributions with minor deviations, parametric statistical analysis was carried out. In particular, two-sample paired *t*-tests were used to analyse the significance of the differences between paired outcome variables from the four different types of strength exercises and two types of loading magnitudes. The level of significance was set at *p* < 0.0125 for all comparisons.

## 3. Results

All results are shown as mean ± standard deviation (SD). Positive values correspond to internal rotation, adduction or flexion angles and the corresponding M_max_. Mean grip width was 88.7 ± 6.7 cm for the bench press exercises.

### 3.1. Shoulder

**Figure 1:**
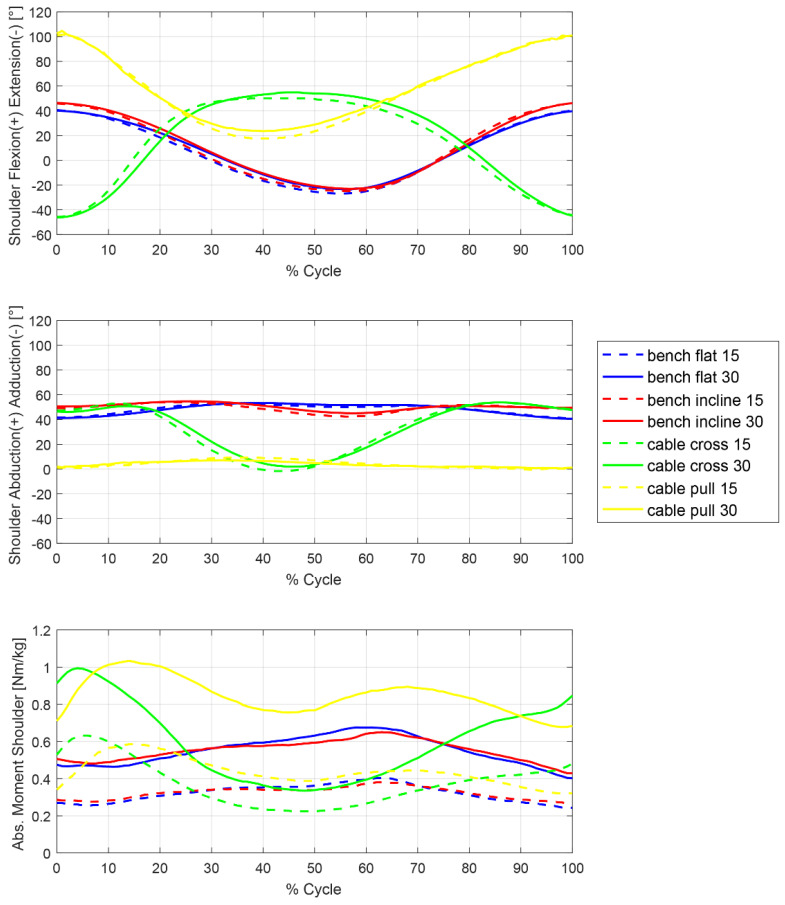
Kinetics and kinematics of the shoulder joint for all exercises performed with 15% BW and 30% BW external load. **Top**: Flexion-extension angle, **middle**: abduction-adduction angle and **bottom**: absolute joint moment, M_abs_.

The mean trajectories of the flexion extension and abduction-adduction joint angles and absolute moments in the shoulder joint during bench press and cable pulley exercises are shown in Figure 1. The results from the statistical comparison of maximum absolute moments are given in Table 1. The flat and inclined bench press exercises showed very similar motion and loading patterns in the shoulder joint. The cross-over exercise presented with substantially different joint angles and loading patterns compared to the pull-over exercise, as well as compared to the bench press exercises, with higher fluctuations of the abduction-adduction angle and absolute joint moment during the exercise cycle. For all exercises, the maximum absolute joint moment was substantially larger with the 30% BW compared to 15% BW loading. Thereby, the maximum absolute shoulder moments were significantly larger for the cable pulley exercises than for the bench press exercises (Table 1). Yet, no significant difference was found between flat versus inclined bench press exercises, as well as between cable pull-over versus cross-over exercises (Table 1). Interestingly, the maximum absolute joint moments during the bench press exercises approximately coincided with the change in movement direction of the upper limb, while maximum joint moments during the cable pulley exercises occurred in the first phase of the exercise cycle (Figure 1).

### 3.2. Elbow

The resulting flexion-extension and supination-pronation RoMs and M_max_ at the elbow joint are given in Table 2. Elbow flexion-extension RoMs were significantly larger during the bench press compared to the cable pulley exercises; elbow supination-pronation RoMs showed opposite behavior, being significantly larger during the cable pulley compared to the bench press exercises. Interestingly, elbow joint RoM was significantly different for the cable cross-over exercise when increasing the load from 15% to 30%; yet, no significant difference was found in elbow M_max_ for the same exercise with increasing load. However, a markedly higher SD in RoM and M_max_ were present for the cable pulley exercises compared to the bench press exercises, suggesting larger variations in joint motion and loading patterns subjects.

### 3.3. Wrist

The resulting flexion-extension and supination-pronation RoMs and M_max_ at the wrist joint are given in Table 3. Flexion-extension and abduction-adduction RoMs of the wrist were significantly larger during the cable pull-over exercise, while the wrist flexion-extension M_max_ was significantly different for the cable cross-over exercise compared to all other exercises. Interestingly, no significant difference in joint RoM was found for any exercise when increasing the load from 15% to 30% BW. Increasing the load also did not result in significantly different abduction-adduction M_max_ for the bench press exercises. However, a large SD in RoM and M_max_ were found for all exercises, suggesting large variations in joint motion and loading patterns between subjects.

## 4. Discussion

The present study reports on the kinematics and kinetics of the upper limbs during four common strength exercises for chest muscles at moderate intensity. Biomechanical assessment of chest muscle strengthening exercises is important for the establishment of training guidelines to minimise injury risks, especially at the shoulder. To the authors’ knowledge, no study has previously compared upper limb kinematics and kinetics between the selected types of exercises.

Biomechanically, the absolute joint moment is counterbalanced by active muscle forces which leads to a reduction of the internally applied joint moments [25]. In resistance training, the absolute joint moment is preferably high during the concentric phase to train the agonist muscle and high during the eccentric phase to train the antagonist muscles, respectively. Yet, a minimisation of the internal joint moments is desirable to avoid overstressing the joint and soft tissue structures [2]. Exercises with changing directions of joint loading should be chosen with care, depending on the training goals, in order to target the intended muscle groups and not their antagonists, as well as to avoid overloading the joint due to a lack of inter-muscular coordination. Given the present results for the shoulder joint, it appears that bench press exercises present with lower and less fluctuating joint loading than cable pulley exercises (Figure 1), likely allowing for more targeted and safe strengthening of the chest muscles.

Previous research suggests that muscle activation patterns during the bench press exercise tend to demonstrate a specificity during moderate-intensity, non-fatiguing exercise execution [8,10]. Thereby, declined bench press was found to induce a greater overall activation of the pectoralis muscles as compared to the inclined bench press. Additionally, it was shown that the anterior and medial deltoid muscles were more active during the bench press performed using free weights compared to the machine [10]. Comparing muscle activity during humeral external rotation with the cable pulley versus the variable resistance machine, it was found that broader ranges of motion with the variable resistance machine led to higher activation of the key movers, especially for heavier loading [6]. Given these results, it is likely that larger fluctuations of joint ranges of motion during cable cross-over exercises in the present study also led to higher activation of the deltoid muscles as joint stabiliser, as well as higher activation of the pectoralis muscle as key mover.

In order to maximise the effect of strength training, it is generally desirable to perform the exercises with a large RoM. This applies for strength training in order to improve, e.g., maximum isometric force, cross-sectional area and inter-/intra-muscular coordination [18,26], as well as for rehabilitation protocols with the aim to regain normal joint function [27]. Additionally, flexibility can be improved if a large RoM during strength training is accomplished [28]. In the present study, the largest flexion-extension RoMs were found at the shoulder joint for the cable cross-over exercise, which also resulted in the largest maximum absolute joint moment (Table 1). Interestingly, the cable cross-over exercise also resulted in significantly larger elbow flexion-extension and supination-pronation RoM when increasing the load from 15% BW to 30% BW. This indicates that the subjects struggled to stabilise the elbow joint during exercise execution with higher loading. The present results suggest that cable pulley exercises may be suited to training intra-muscular coordination across the upper limbs. Yet, care has to be taken when performing cable cross-over exercise to ensure secure stabilisation of the joints during exercise execution, in particular in the rehabilitation setting, and proper supervision by a professional instructor is highly recommended. In the present study, BW was used for the assignment of loading. In the athletic setting, however, it is more common to use the so-called one-repetition maximum (1RM) as a key indicator of an individual’s dynamic strength [29]. Using the 1RM instead of BW might have facilitated the comparison of the present results with the literature. However, the direct assessment of 1RM is time-consuming and depends on the athlete’s experience, motivation and fatigue, with risk of musculoskeletal injury due to maximum loading [30]. As an explorative study, the main goal here was to, firstly, characterise joint kinematics and kinetics at moderate intensities to improve training guidelines for injury prevention and rehabilitation settings. In the future, recent advances in smartwatch-based technologies hold great potential to indirectly assess an individual’s 1RM via linear regression techniques without exposing subjects to maximum loading [29]. The future use of smartwatch-based technologies to complement the present study protocol may also allow direct assessment of barbell force based on the acceleration for deriving joint moments during the bench press exercise, without the need to use force platforms and a bottom-up approach.

The key limitation of the present study is the simplified representation of the shoulder joint for biomechanical analysis. In particular, shoulder kinematics were described as the movement of the upper arm relative to the thorax without considering the intricate movement of the shoulder girdle. For small joint ranges of motion, such a simplification is considered valid. However, during the final part of the bench press exercise, protraction of the scapula is fundamental for reaching the final position of the barbell and, thus, substantially contributes to shoulder joint kinematics and RoM. Anatomically, the glenohumeral joint is a ball-and-socket joint with three rotational degrees of freedom between the scapula and the humerus, and the mobility of the shoulder complex is further increased by the sternoclavicular, acromioclavicular and scapulothoracic joints. This complex range of movement challenges the analysis of shoulder kinematics during dynamic exercises using optical motion capture because of skin movement artefacts [22]. Further work should concentrate on refining optical marker sets and fitting techniques to reduce skin movement artefacts for the assessment of shoulder kinematics. Thereby, the clavicle and the scapula are ideally defined as separate segments to investigate the intricate movement across the shoulder girdle. This would not only be important for developing targeted guidelines for chest strength training but also for orthopaedic and musculoskeletal research in general.

Detailed biomechanical analyses of both bench press and cable pulley exercises, including muscle activation patterns, muscle force estimation and internal joint loading at the shoulder joint, are recommended to gain further insights into the injury risks associated with each type of exercise. Here, a refinement of study protocol based on good practice rules for the assessment of the force–velocity relationship during strength training is advisable [31]. In particular, subjects may naturally express less effort in lifting lower loads if lifting at maximum velocity is not ensured. Thus, heavier loads may have been lifted with the same or even higher velocities compared to lighter loads, which may have biased the present results. Furthermore, it is advisable to assess upper limb kinematics and kinetics during the bench press exercise with different inclination angles in order to refine training recommendations. In particular, further research may examine whether joint motion and joint loading significantly change with bench inclination and for subjects with different body constitution.

## 5. Conclusions

A combination of bench press and cable pulley exercises, starting with smaller loads and guided supervision, is recommended to achieve functional training of the chest muscles to stabilise the upper limb and avoid overstressing the joint and soft tissue structures. Lower and less fluctuating joint moments during bench press exercises imply more targeted muscle strengthening with reduced need for inter-muscular control and coordination compared to cable pulley exercises. Care has to be taken when performing cable cross-over exercises to ensure proper stabilisation of the joints during exercise execution and avoid joint overloading.

## Figures and Tables

**Table 1:** Mean values and SD for maximum absolute joint moment (M_max_) calculated across all 20 subjects. The two bottom rows associated with (*) indicate significant differences (*p* < 0.0125) of the particular exercise with respect to flat bench press (bf), incline bench press (bi), cable cross-over (cc) and/or cable pull-over (cp) with equal load, as well as significant differences (*p* < 0.0125) of the particular exercise with respect to 15% BW or 30% BW, respectively.

Shoulder Moment [Nm/kg]		Bench_Flat (bf)		Bench_Incline (bi)		Cable_Cross (cc)		Cable_Pull (cp)	
		15% BW	30% BW	15% BW	30% BW	15% BW	30% BW	15% BW	30% BW
**absolute**	Mean ± SD	0.442	0.760	0.418	0.712	0.650	1.026	0.611	1.09
		±0.046	±0.079	±0.041	±0.080	±0.113	±0.192	±0.086	±0.155
	*	cc, cp	cc, cp	cc, cp	cc, cp	bf, bi	bf, bi	bf, bi	bf, bi
		30	15	30	15	30	15	30	15

**Table 2:** Mean values and SD for elbow joint flexion-extension and supination-pronation RoM and M_max_ calculated across all 20 subjects. The rows associated with (*) indicate significant differences (*p* < 0.0125) of the particular exercise with respect to flat bench press (bf), incline bench press (bi), cable cross-over (cc) and/or cable pull-over (cp) with equal load, as well as significant differences (*p* < 0.0125) of the particular exercise with respect to 15% BW or 30% BW, respectively.

Elbow			Bench_Flat (bf)		Bench_Incline (bi)		Cable_Cross (cc)		Cable_Pull (cp)	
			15% BW	30% BW	15% BW	30% BW	15% BW	30% BW	15% BW	30% BW
**RoM (°)**	Flexion-Extension	Mean ± SD	79.5	76.2	84.3	81.4	21.8	32.5	23.3	30.9
			±6.2	±6.4	±6.4	±6.2	±10.9	±15.2	±6.5	±8.8
		*	cc, cp	cc, cp	cc, cp	cc, cp	bf, bi	bf, bi	bf, bi	bf, bi
							30	15	30	15
	Supination-Pronation	Mean ± SD	6.5	6.4	7.0	7.2	11.6	15.6	21.6	19.5
			±1.9	±1.7	±2.8	±2.8	±6.1	±10.6	±5.3	±4.6
		*	cc, cp	cc, cp	cc, cp	cc, cp	bf, bi, cp	bf, bi, cp	bf, bi, cc	bf, bi, cc
							30	15		
**M_max_ (Nm/kg)**	Flexion-Extension	Mean ± SD	0.092	0.165	0.112	0.201	−0.042	−0.059	−0.004	0.004
			±0.018	±0.041	±0.023	±0.043	±0.011	±0.017	±0.067	±0.110
		*	cc, cp	cc, cp	cc, cp	cc, cp	bf, bi, cp	bf, bi, cp	bf, bi, cc	bf, bi, cc
			30	15	30	15				
	Supination-Pronation	Mean ± SD	0.016	0.030	0.016	0.030	−0.008	−0.017	0.165	0.237
			±0.004	±0.008	±0.004	±0.008	±0.007	±0.012	±0.032	±0.069
		*	cp	cp	cp	cp	cp	cp	bf, bi, cc	bf, bi, cc
									30	15

**Table 3:** Mean values and SD for wrist joint flexion-extension and abduction-adduction RoM and M_max_ calculated across all 20 subjects. The rows associated with (*) indicate significant differences (*p* < 0.0125) of the particular exercise with respect to flat bench press (bf), incline bench press (bi), cable cross-over (cc) and/or cable pull-over (cp) with equal load, as well as significant differences (*p* < 0.0125) of the particular exercise with respect to 15% BW or 30% BW, respectively.

Wrist			Bench_Flat (bf)		Bench_Incline (bi)		Cable_Cross (cc)		Cable_Pull (cp)	
			15% BW	30% BW	15% BW	30% BW	15% BW	30% BW	15% BW	30% BW
**RoM (°)**	Flexion-Extension	Mean ± SD	11.9	11.0	14.0	12.4	21.1	18.7	34.4	37.1
			±4.0	±2.7	±3.7	±3.9	±8.6	±7.8	±13.8	±11.9
		*	cc, cp	cc, cp	cc, cp	cc, cp	bf, bi, cp	bf, bi, cp	bf, bi, cc	bf, bi, cc
							
	Abduction-Adduction	Mean ± SD	9.8	9.8	10.6	10.4	11.7	12.1	33.8	31.1
			±2.5	±2.2	±2.1	±1.8	±6.0	±4.1	±14.3	±11.6
		*	cp	cp	cp	cp	cp	cp	bf, bi, cc	bf, bi, cc
							
**M_max_ (Nm/kg)**	Flexion-Extension	Mean ± SD	−0.015	−0.032	−0.017	−0.035	0.025	0.046	−0.017	−0.054
			±0.005	±0.012	±0.006	±0.011	±0.009	±0.012	±0.031	±0.034
		*	cc	cc, cp	cc	cc, cp	bf, bi, cp	bf, bi, cp	cc	bf, bi, cc
			30	15	30	15	30	15	30	15
	Abduction-Adduction	Mean ± SD	0.011	0.019	0.010	0.018	−0.027	−0.040	0.021	0.053
			±0.005	±0.010	±0.005	±0.009	±0.010	±0.015	±0.021	±0.028
		*	cc	cc, cp	cc	cc, cp	bf, bi, cp	bf, bi, cp	cc	bf, bi, cc
							30	15	30	15

## Data Availability

Not applicable.

## Supplementary Materials

The following supporting information can be downloaded at: https://www.mdpi.com/article/10.3390/sports10020019/s1, Additional File 1—Instructed Execution of the Exercises; Additional File 2—Basic Motion Tasks; Additional File 3—Segment and Joint Coordinate System Definitions.
