# Supplementary material for: Chest Exercises: Movement and Loading of Shoulder, Elbow and Wrist Joints
Source: Sports (Basel). 2022 Jan 31;10(2):19. doi: 10.3390/sports10020019 (PMC8877248; doi:10.3390/sports10020019)
Supplement: Supplementary file 1 [file sports-10-00019-s001.zip › sports-1544701 - Supplementary Material (Updated).pdf]

Additional Files

Additional File 1 – Instructed Execution of the Exercises

The measured exercises were the flat and incline bench press as well as cross-over and pull-over on the cable pulley. All subjects received the same instructions for the execution of the exercises. They were allowed to perform the exercises in their preferred speed and style but had to stick to certain fundamental directives, which are explained in detail below.

Flat Bench Press

During the flat bench press subjects were instructed to lie on the flat weight bench with angled knee joints, putting their feet down on the floor beside the bench. Shorter subjects who could not reach the ground were allowed to place their feet on the bench legs as the height of the bench was not adjustable. Subjects had to hold the barbell with both hands at given grip width, which was marked on the barbell. The initial position was with the arms stretched over the chest with nearly but not fully extended elbow joints. The barbell was then lowered towards the chest until almost touching it. Afterwards the barbell was pushed upwards again until the initial position was reached again. Head, hip and thighs had to remain stable on the bench during the entire movement. Initial and final position of the movement are shown in Figure S1.

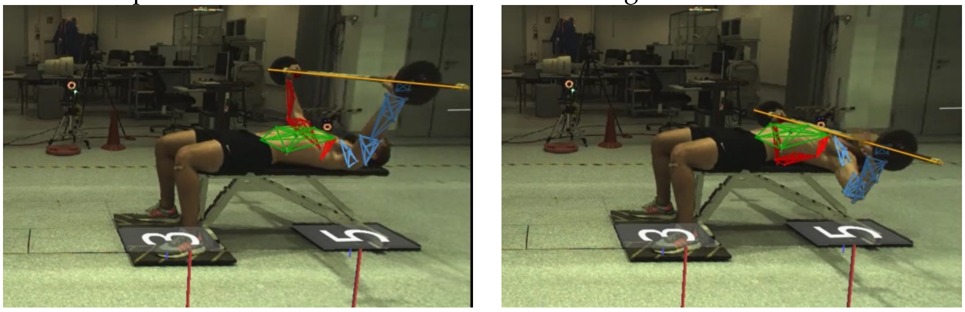

Figure S1: Initial (left) and final (right) position of the flat bench press.

Inclined Bench Press

Instructions for the inclined bench press exercise were corresponded with those for the flat bench press except the bench was inclined by 45° for this exercise. Subjects again started with the arms extended, holding the barbell above the chest before lowering it down towards the chest until reaching the final position just before the barbell touches the chest and bringing it back up to the initial position. Initial and final position of the movement are shown in Figure S2.

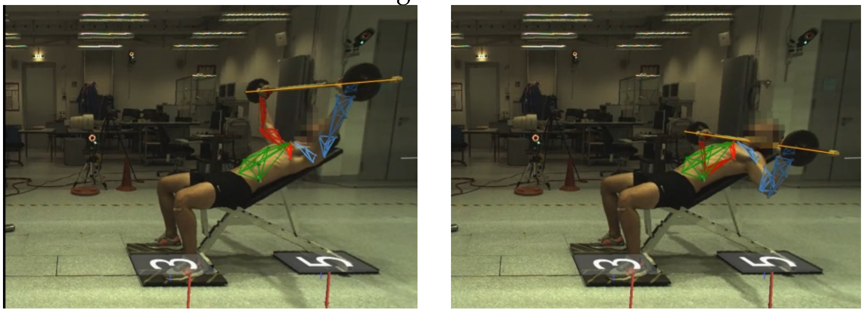

Figure S2: Initial (left) and final (right) position of the incline bench press.

Cable Cross-Over

The height of the cable pulley station was adjusted so the cable was on the same level as the subject's head. For the cross-over exercise subjects had to stand between the two

towers of the cable pulley station in lunge position, whereby it was not prescribed which leg had to be in front, with the upper body bent forward. The load was distributed evenly on both towers of the cable pulley station. To begin with, the arms were stretched sideways with the elbow joints slightly bent and were then brought together in front of the body. Afterwards the arms were moved back again to the initial starting position. During the whole movement the arms and the cable had to be arranged in the same plane. Initial and final position of the movement are shown in Figure S3.

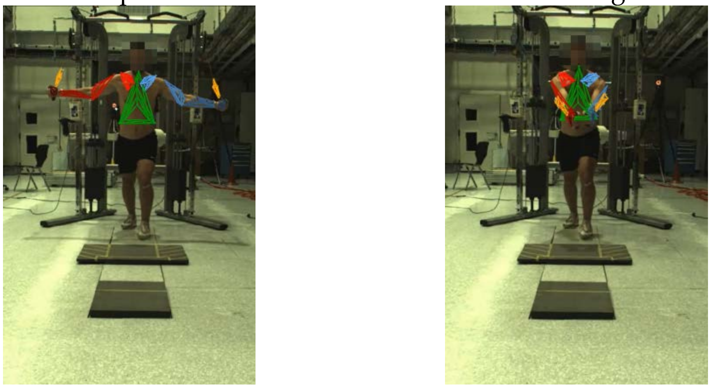

Figure S3: Initial (left) and final (right) position of the cross-over exercise.

Cable Pull-Over

The height of the cable pulley station was as well as for the cross-overs the height of the subject. Other than during the cross-over exercise only one tower of the cable pulley station was used. The subjects stood in front of the training device the feet about shoulder width apart with the knees bent and the trunk slightly bent forward. In the initial position the subjects had the arms stretched forward, holding the rope. The arms had to be pulled down towards the thighs and had to be pulled apart so the hands were at the same level as the thighs at the end of the pulling movement. To complete the movement the subjects had to bring the arms back together and up to the initial position. Initial and final position of the movement are shown in Figure S4.

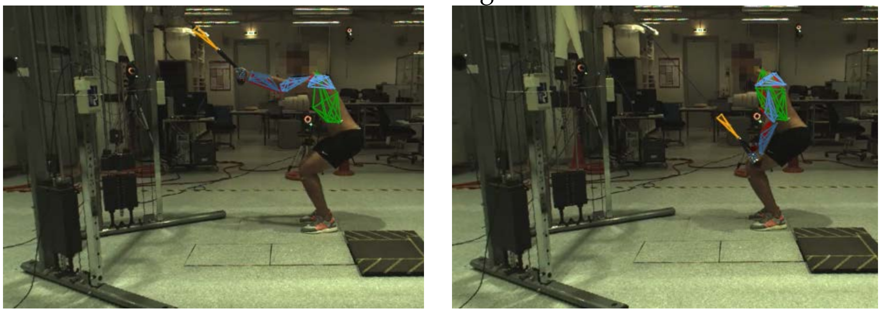

Figure S4: Initial (left) and final (right) position of the pull-over exercise.

Additional File 2 – Basic Motion Tasks

BMT Shoulder

**Sagittal Flexion-Extension**	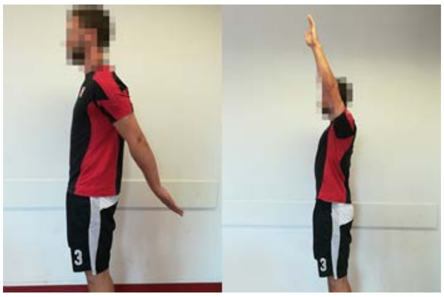

**Frontal Abduction-Adduction**	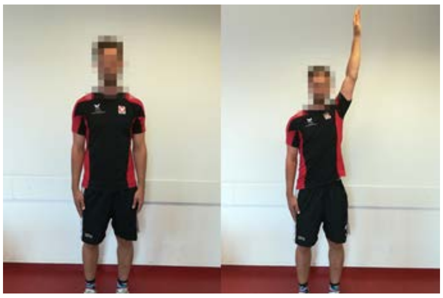
**Transversal Abduction-Adduction**	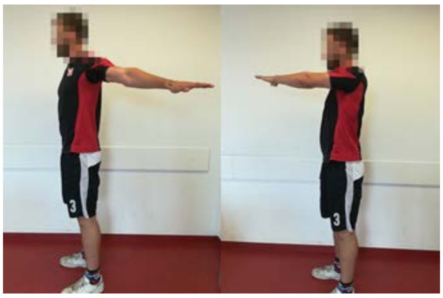
**Circumduction**	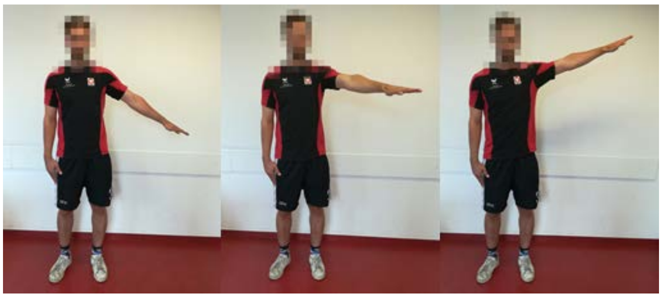

BMT Elbow

**Flexion-Extension**	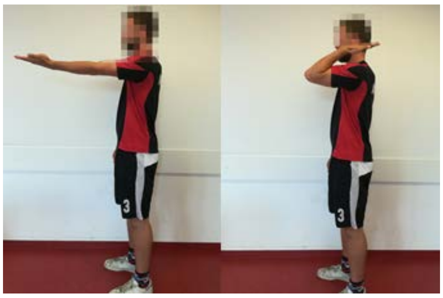
**Supination-Pronation**	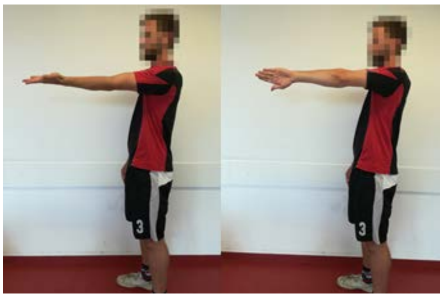

BMT Wrist

**Flexion-Extension**	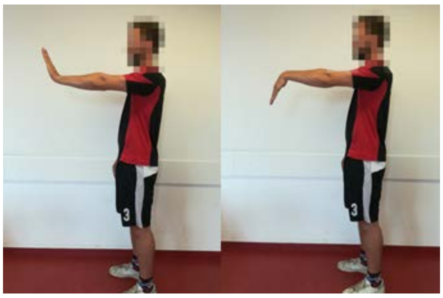

**Abduction-Adduction**	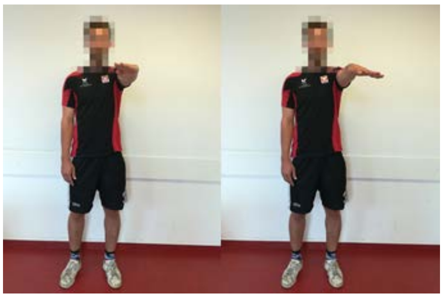
**Circumduction**	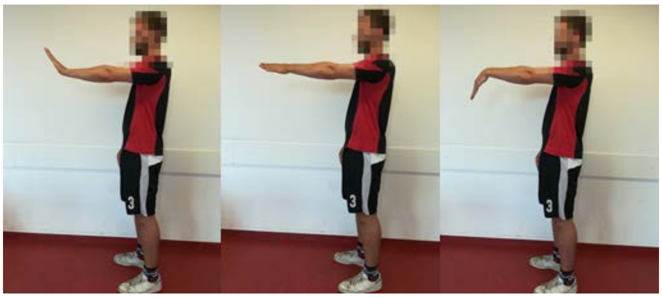

<u>*Additional File 3 – Segment and Joint Coordinate System Definitions*</u>

	Segment coordinate systems
	**Segment coordinate system torso**
**e_{to1} mediolateral axis**	e_{to1} is perpendicular to the plane formed by the markers STCR, STCA, SPC7 and SPT8, pointing to the right.
e_{to3} longitudinal axis	e_{to3} is perpendicular to e_{to1} and lies in the plane formed by e_{to1}, the middle point of the connecting line between STCR and SPC7 and the middle point of the connecting line between STCR and SPT8, pointing towards cranial.
e_{to2} anteroposterior axis	e_{to2} is perpendicular to e_{to1} and e_{to3}, pointing to the front.
	**Segment coordinate system upper arm**
e_{ua3} longitudinal axis	e_{ua3} corresponds to the line connecting GHJC and the midpoint of RTLC/LTLC and RTMC/LTMC, pointing towards GHJC.
e_{ua2} anteroposterior axis	e_{ua2} is perpendicular to the plane formed by RTLC/LTLC, RTMC/LTMC and GHJC pointing to the front.
e_{ua1} mediolateral axis	e_{ua1} is perpendicular to e_{ua2} and e_{ua3}, pointing to the right.
	**Segment coordinate system forearm**
e_{fa3} longitudinal axis	e_{fa3} corresponds to the connecting line between EJC and WJC, pointing towards EJC.
e_{fa1} mediolateral axis	e_{fa1} is perpendicular to e_{fa3} and lies in the plane formed by the markers RWRA/LWRA, RWRB/LWRB and EJC, pointing to the right.
e_{fa2} anteroposterior axis	e_{fa2} is perpendicular to e_{fa1} and e_{fa3}, pointing to the front.
	**Segment coordinate system hand**
e_{ha2} anteroposterior axis	e_{ha2} is perpendicular to the plane formed by the four hand markers RCM5/LCM5, RCM2/LCM2, RDM5/LDM5 and RDM2/LDM2, pointing to the front.
e_{ha3} longitudinal axis	e_{ha3} is perpendicular to e_{ha2} and lies in the plane of e_{ha2} and the connecting line between the middle points of RDM2/LDM2 to RDM5/LDM5 and RCM2/LCM2 to RCM5/LCM5, pointing towards the middle point of RCM2/LCM2 to RCM5/LCM5.
e_{ha1} mediolateral axis	e_{ha1} is perpendicular to e_{ha2} and e_{ha3}, pointing to the right.
	Joint coordinate systems
	**Joint coordinate system shoulder**
**e_{SH1}**	e_{SH1} is fixed at the proximal segment (torso) and corresponds to e_{to1}.

**mediolateral axis**	
e_{SH3}<br>longitudinal axis	e_{SH3} is fixed at the distal segment (upper arm) and corresponds to e_{ua3}.
e_{SH2}<br>anteroposterior axis	e_{SH2} is perpendicular to e_{SH1} and e_{SH3} (floating axis).
**Joint coordinate system elbow**	
e_{EL1}<br>mediolateral axis	e_{EL1} is fixed at the proximal segment (upper arm) and corresponds to e_{ua1}.
e_{EL3}<br>longitudinal axis	e_{EL3} is fixed at the distal segment (forearm) and corresponds to e_{fa3}.
e_{EL2}<br>anteroposterior axis	e_{EL2} is perpendicular to e_{EL1} and e_{EL3} (floating axis).
**Joint coordinate system wrist**	
e_{WR1}<br>mediolateral axis	e_{WR1} is fixed at the proximal segment (forearm) and corresponds to e_{fa1}.
e_{WR3}<br>longitudinal axis	e_{WR3} is fixed at the distal segment (hand) and corresponds to e_{ha3}.
e_{WR2}<br>anteroposterior axis	e_{WR2} is perpendicular to e_{WR1} and e_{WR3} (floating axis).
